# Analysis of the correlation between climatic variables and Dengue cases in the city of Alagoinhas/BA

**DOI:** 10.1038/s41598-023-34349-8

**Published:** 2023-05-09

**Authors:** Marcos Batista Figueredo, Roberto Luiz Souza Monteiro, Alexandre do Nascimento Silva, José Roberto de Araújo Fontoura, Andreia Rita da Silva, Carolina Aparecida Pereira Alves

**Affiliations:** 1 Departamento de Ciências Exatas e da Terra II, Universidade do Estado da Bahia, Alagoinhas, BA Brasil; 2 Centro Universitário SENAI CIMATEC, Salvador, BA Brasil; 3 Departamento de Engenharias e Computação, Universidade Estadual de Santa Cruz, Ilhéus, BA Brasil

**Keywords:** Applied mathematics, Computational biology and bioinformatics, Data processing

## Abstract

The Aedes aegypti mosquito is the main vector of dengue and is a synanthropic insect and due to its anthropophilic nature, it has specific reproductive needs. In addition to that, it also needs tropical regions that provide climate-prone conditions that favor vector development. In this article, we propose the cross-correlation analysis between the climatic variables air temperature, relative humidity, weekly average precipitation and dengue cases in the period from 2017 to early 2021 in the municipality of Alagoinhas, Bahia, Brazil. To do so, we apply the trend-free cross-correlation, $\rho DCCA$, being a generalization of the fluctuation analysis without trend, where we calculate the cross correlation between time series to establish the influence of these variables on the occurrence of dengue disease. The results obtained here were a moderate correlation between relative humidity and the incidence of dengue cases, and a low correlation for relative air temperature and precipitation. However, the predominant factor in the incidence of dengue cases in the city of Alagoinhas is relative humidity and not air temperature and precipitation.

## Introduction

Dengue is a public health problem affecting several municipalities in Brazil^1–3^. The transmitting agent is the female Aedes aegypti mosquito, and its transmission occurs through its bite, passing the virus to the host. The individual may develop the disease or not^4^. Dengue affects thousands of people each year in several countries, including Brazil and other regions of Latin America^5–7^.

According to data from the Brazilian Ministry of Health, the country recorded more than 1.5 million cases of dengue in 2019, with 782 deaths related to the disease^8^. In addition, the region of Latin America and the Caribbean is considered the most affected by dengue, with a total of 2.2 million cases reported in 2019^9^. The epidemiological situation of dengue in Brazil and Latin America is complex and challenging, as the disease is endemic in many areas and transmission can be influenced by factors such as climate change, urbanization and population migration^2^. Dengue control requires a multifaceted approach that involves prevention measures, early detection and adequate treatment of cases, thus understanding how climate variations affect this transmission is of paramount importance for understanding the vectors^4,10^.

All four serotypes can lead to severe dengue in the first infection, but more frequently after the second or third infection, with no proven statistical difference whether after the second or third infection^11^; there is a proportion of cases that have the subclinical infection, that is, they are exposed to the infective bite of the *Aedes aegypti* mosquito but do not clinically present the disease, although they are immune to the serotype with which they were infected; this occurs in 20–50% of infected people^12^.

The second infection by any dengue serotype is predominantly more severe than the first, regardless of serotypes and their sequence. However, serotypes DENV2 and DENV3 are considered more virulent^11^.

The four serotypes of the dengue virus (DENV1, DENV2, DENV3, and DENV4), it’s which makes vaccine development particularly challenging^13,14^. Despite ongoing efforts to develop a vaccine, no vaccine has been developed yet that is effective against all four serotypes of dengue^15,16^. This is due to the complex nature of the virus, which can mutate rapidly and create new strains, making it difficult to target with a single vaccine. Furthermore, there are concerns about vaccine-induced enhancement of disease, which has further complicated the development of a safe and effective vaccine. As a result, prevention strategies such as mosquito control and personal protective measures remain the primary methods for reducing the incidence of dengue fever^16–18^.

They are transmitted to humans by species of mosquitoes of the genus *Aedes*, with *Aedes aegypti* being the main vector^12^.

The presence of Aedes Aegypti in urban centers is directly related to climatic conditions and public sanitation, with climatic variables playing a significant role in the distribution of the mosquito^10^. Currently several phenomena have caused global variations in climatic conditions. One example is projections that indicate an increase between 1.8 and 4 $^\circ$C^19,20^ in temperature. In Brazil temperatures are expected to rise between 1.8 and 4 $^\circ$C^21–23^.

Dengue is the most prevalent urban arbovirus in the Americas, mainly in Brazil^24,25^. It is a febrile illness that has been of great importance in public health in recent years^26–28^. Dengue virus (DENV) is an arbovirus transmitted by the bite of the female Aedes aegypti mosquito and has four different serotypes (DENV-1, DENV-2, DENV-3 and DENV-4).

Dengue fever affects people of all ages, but adults and young people have been the most affected by the disease since the introduction of the virus in Alagoinhas. However, as of 2006, some states showed the recirculation of serotype DENV2 after a few years of predominance of serotype DENV3^29,30^. This scenario led to an increase in the number of cases of severe forms and hospitalizations in children, mainly in the Northeast of the country^30–32^.

These epidemics were characterized by a pattern of reduced severity for children, who accounted for more than 50% of hospitalized patients in municipalities with the largest population^33^. Even in municipalities with a smaller population, more than 25% of patients hospitalized for dengue were children, which highlights that the whole country has been suffering, in a similar way, these changes in the profile of the disease^34^. Despite the reduction in the severity of the disease, dengue remains a public health problem, especially in municipalities with low population, such as Alagoinhas/BA^35^, where this research was carried out.

In this study, we aimed to analyze the cross-correlation between the climatic variables air temperature, relative air humidity and average weekly precipitation and dengue cases in the municipality of Alagoinhas, Bahia, Brazil.

To carry out this work, combined data from two sources were used. The first from the meteorological station in the city of Alagoinhas, Bahia, maintained by the National Institute of Meteorology and epidemiological data from the FioCruz Dengue Observatory^36^. These raw data are collected at latitude: $-12.28$, longitude: $-38.55$, altitude: 212 m.

In the datasets obtained, we have separated the columns of data corresponding to dengue cases, air temperature, humidity relative air and weekly average precipitation. We used these data to calculate trendless cross-correlation coefficients, $\rho DCCA$^37^, for air temperature, relative air humidity and precipitation in relation to confirmed cases of dengue, in order to establish the influence of these variables on the occurrence of the disease. We plotted the graph of the $\rho DCCA$s of the collected variables in order to study the influence of these variables on dengue cases. The scales were determined automatically by the MaiaStatistics software^38^ which follows the algorithm defined by Peng^39^ and Zebende^37^.

The analysis revealed a moderate correlation between relative humidity and the incidence of dengue cases in the municipality of Alagoinhas between the years 2017 and 2020 and a low correlation for relative air temperature and precipitation.

## Materials and methods

Alagoinhas is a Brazilian municipality in the state of Bahia, located in the North Coast and Agreste region of Bahia. Its area is 718.089 square kilometers and its population in 2020 was 153.023 inhabitants, therefore having a population density of 210.05 inhabitants per square kilometer. It is limited to the north by the municipality of Inhambupe, to the south by the municipality of Catu, to the east by the municipality of Araçás, to the west by the municipality of Aramari, to the northeast by the municipality of Entre Rios and to the southwest by the municipality of Teodoro Sampaio. The municipality is crossed by the BR-101, which goes towards the State of Sergipe. This municipality was chosen because it presents a significant increase in dengue cases in the region.

### The dataset

The study carried out is a computational ecological type based on time series with design between the years 2017 and 2021. Data on the number of confirmed cases were obtained from InfoDengue^36^, which is an alert system for arboviruses based on hybrid data generated through the integrated analysis of data mined from the social web and climatic and epidemiological data.

The meteorological variables, Temperature, Relative Humidity and Precipitation were obtained by a station located in the municipality that collects this data daily^40^.

**Figure 1:**
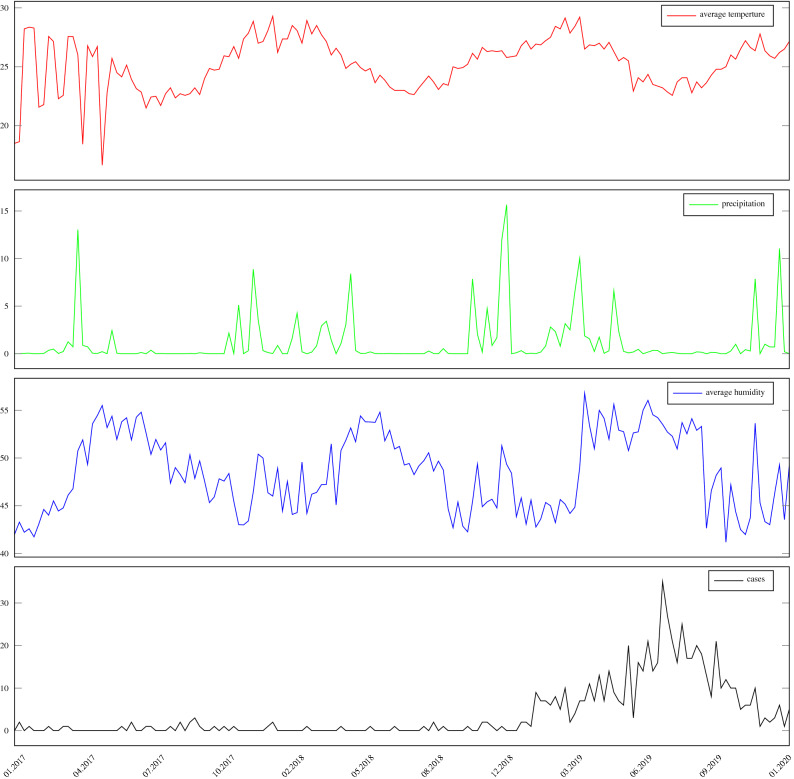
The figure illustrates the difficulty of perceiving a correlation between variables without using advanced techniques such as $\rho$dcca.

Data relating to the epidemiological condition of the municipality are reported weekly while climate variables are available daily. So that the time series have the same seasonality, we calculate the weekly average of these variables. Figure 1 shows these data and reveals that the municipality has a temperature with little fluctuation, sparse rain showers and a large variation in relative humidity.

An important factor is the absence of climate data between 2021 and 2022 due to the coronavirus pandemic, since the weather station is manually operated. For this reason, the research was limited to data from 2017 to early 2021.

### How calculate the coefficient

The unbiased cross-correlation coefficient, $\rho$DCCA^37^ is used to determine whether two time series *y* e $y'$ present negative, null or positive correlation in scale *n*, presenting values in the range $-1\leqslant \rho DCCA \leqslant 1$. We calculated $\rho$ DCCA(*n*), for an *n* scale using Eq. (1)

1 $$\begin{aligned} [!htb] \rho DCCA(n)=\frac{F^2_{DCCA}(n)}{F_{DFA_y}(n) \times F_{DFA_y'}(n) } \end{aligned}$$

where $F^2_{DCCA}(n)$ is the DCCA^41^ for time series *y* e $y'$ on scale *n*, where $F_{DCCA_y}(n)$ is the DFA^39^ for time series *y* on scale *n* e $F_{DCCA_{y'}}(n)$ is the DFA for the time series $y'$ at *n* scale.

To calculate the value of $F^2_{DCCA}(n)$ we used Eq. (2) where *N* corresponds to the number of elements in each time series, *n* to the size of the scale (box) and $f^2_{DCCA}(n.i)$ corresponds to the mean value of the products of the residues of each series and each range, given by Eq. (3)

2 $$\begin{aligned}{} & {} f^2_{DCCA}(n)\equiv \left( N-n\right) ^{-1}\times \sum _{i=1}^{N-n}f^2_{DCCA}(n,i) \end{aligned}$$

3 $$\begin{aligned}{} & {} f^2_{DCCA}(n,i)\equiv \frac{1}{n+1}\times \sum _{k=1}^{1+n}\left( R_k- {\tilde{R}}_{k,i} \right) \times \left( R'_k- {\tilde{R}}{'_{k,i} }\right) \end{aligned}$$

In Eq. (3) $R_k$ is the *y* series integrated into the *k* box given by Eq. (4) and ${\tilde{R}}_{k,i}$ is the value of the linear fit, using the method of least squares, for the *y* series at point *i* and $R'_k$ and the $y'$ series integrated into box *k*, given by Eq. (5), while $R'_{k,i}$ is the value of the linear fit, by least squares, for the series $y'$, at point *i*.

4 $$\begin{aligned}{} & {} R_k\equiv \sum _{i=1}^ky_i \end{aligned}$$

5 $$\begin{aligned}{} & {} R'_k\equiv \sum _{i=1}^ky'_i \end{aligned}$$

To calculate the $F_{DFA}(n)$ of each series in each scale (box 0) *n* we use Eq. (6), which corresponds to the value of the square root of the squared residues, in each box, where *y*(*k*) is the value of the series in the point *k* and $y_n(k)$ is the value of the linear fit, using the method of least squares, for the *y*-series, at point *k*.

6 $$\begin{aligned} F_{DFA}(n)=\sqrt{\frac{1}{n}\sum _{k=1}^N\left[ y(k)-y_n(k)\right] ^2} \end{aligned}$$

We used Eqs. (1), (2), (3), (4), (5), (6) and the MaiaStatistics software^38^ to calculate the $\rho$DCCA value.

## Results

The city of Alagoinhas is located close to the equator ($12^\circ \, 8'\, 9''$ S $38^\circ \, 25'\, 8''$ W), which makes the seasons difficult to be characterized, presenting a climate between 2017 and 2021 with low annual precipitation. The remarkable interannual variability of rainfall, associated with low total annual rainfall values is a reflection of the macroclimate of the Northeast region of Brazil and one of the main factors for the occurrence of “drought” events, characterized by a sharp reduction in total seasonal rainfall during the rainy period.

The interannual variability of rainfall in the city is associated with variations in Sea Surface Temperature (SST) patterns over the tropical oceans, which affect the position and intensity of the Intertropical Convergence Zone (ITCZ) over the Atlantic Ocean^42^.

**Figure 2:**
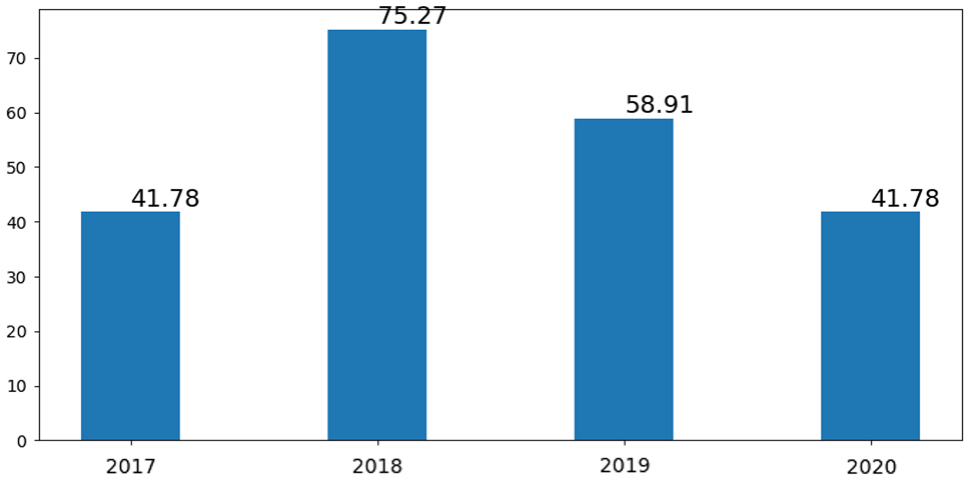
The figure shows the rainfall variation in the municipality of Alagoinhas between 2017 and 2020.

The city of Alagoinhas has a measured rainfall described in Fig. 2, which demonstrates that rainfall comes seasonally, abruptly and in short periods of time. Temperatures, as shown in Fig. 1, shows a little variation, with a maximum recorded of 35 $^\circ$C and a minimum of 20 $^\circ$C, which can occur during all seasons of the year, with greater variations in the hottest months of the year, January and March, and the mildest between June and September.

The average relative humidity is 48%, with a maximum of 76% and a minimum of 20%. These data indicate stability in the humidity index in the months of July to September while in the others there are both high or low peaks.

The period of study of dengue cases in the municipality was 4 years, in which 1.483 confirmed cases were registered, which indicates an incidence in the population, in the described period, of about 35%, according to the methodology described in the technical note on dengue from the Council National Health Secretariats on Dengue Incidence Rate.

Observing Fig. 1, we do not perceive a direct relationship of growth and decrease between the time series of registered cases of dengue and the climatic variables. Thus, in this study we seek a deeper understanding of the cross-relationship between these series using the cross-correlation coefficient $\rho$DCCA.

**Figure 3:**
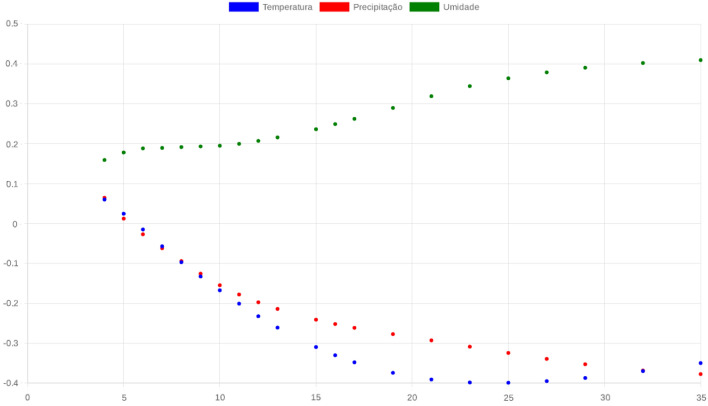
The $\rho$DCCA between temperature, humidity and precipitation and dengue cases show that there is a positive correlation between humidity and dengue cases and a negative correlation with temperature and precipitation.

**Figure 4:**
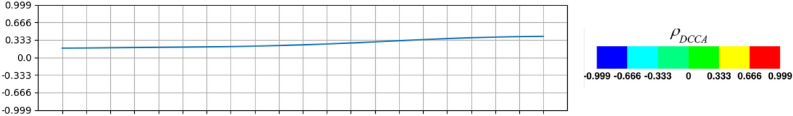
Behavior of $\rho$DCCA in relation to relative humidity showing the positive correlation between two params.

Figures 3 and 4 shows this result, which shows an average correlation between relative humidity and confirmed cases of dengue and a low correlation between the variables relative air temperature and precipitation. Although several authors^43^ claim that the variables air temperature and precipitation have a direct influence on the incidence of dengue, in the city of Alagoinhas these variables are a little less relevant than humidity.

**Table 1:** Detrended cross-correlation intervals.

Condition	$\rho$DCCA
Weak	$\pm 0.000\rightarrow \pm 0.333$
Medium	$\pm 0.333 \rightarrow \pm 0.666$
Strong	$\pm 0.666 \rightarrow \pm 0.999$

According to the six levels described in Table 1 (three positive and three negative) in this article, we can associate the color with a range of $\rho$DCCA. In our case, the $\rho$DCCA value was positive for the humidity being in the yellow range. This perception is not observed for the other variables that remained in the range between blue and light green.

For Alagoinhas, and the data obtained (shown in Table 2), we observed a localized level of anti-correlation (blue) and $n\approx 2$. In the windows $n\approx 32$, an average correlation for humidity is clearly perceived while weak for temperature and precipitation.

**Table 2:** Climate data for Alagoinhas/BA 2017 to 2020.

	Temp. Min ($^\circ$C)	Temp. Max ($^\circ$C)	Temp. Med ($^\circ$C)	Air humidity Max (%)	Air humidity Min (%)	Air humidity mean (%)	precipitation (mm)
Year 2017							
Mean	19.821429	28.785714	24.303571	76.895697	19.913402	48.404549	0.803516
Std	2.397123	3.393907	2.821127	7.577282	3.877057	4.015551	2.298717
Min	13.285714	19.428571	16.642857	61.857143	0.000000	41.740387	0.000000
Max	23.285714	34.714286	28.857143	89.571429	27.009996	55.504998	13.042857
Year 2018							
Mean	21.019231	29.934066	25.476648	77.218930	19.445824	48.332377	1.447473
Std	1.576648	2.271042	1.764026	7.281375	2.632054	3.408632	3.122513
Min	17.571429	26.428571	22.642857	65.000000	15.038893	42.266736	0.000000
Max	24.000000	35.285714	29.285714	91.714286	23.931910	54.809667	15.654286
Year 2019							
Mean	21.184066	30.203297	25.693681	78.599152	19.560227	49.079689	1.132912
Std	1.548298	2.358156	1.799100	9.793907	3.267813	4.752521	2.129416
Min	18.428571	26.000000	22.571429	64.000000	13.439754	41.184116	0.000000
Max	24.571429	34.571429	29.214286	92.428571	28.582917	56.791616	10.028571
Year 2020							
Mean	20.107143	29.508242	24.807692	43.260871	26.979886	35.120379	1.525330
Std	1.646385	2.231036	1.803166	26.108015	9.533392	13.130460	2.850252
Min	13.571429	25.571429	19.571429	10.170849	7.239520	15.094769	0.000000
Max	22.857143	33.285714	27.428571	93.728746	48.572965	56.858965	11.055714

## Conclusion

In this article, we seek to understand the correlation between the time series related to relative humidity, air temperature, precipitation and confirmed cases of dengue in the municipality of Alagoinhas/BA from 2017 to 2021. We compared weekly data and observed the existence of an inverse correlation between air temperature and precipitation and a direct correlation between humidity and dengue cases. We apply the detrended cross-correlation coefficiente, $\rho DCCA$, to measure this two-by-two correlation of these non-stationary series. The interesting fact observed is that, different from what is stated in the literature, the predominant factor in the incidence of dengue cases in the city of Alagoinhas is the relative humidity of the air and not the air temperature and precipitation.

## Data Availability

The data and materials used in this research are available upon request. Interested parties may contact me at mbfigueredo@uneb.br to obtain a copy of the data and materials. Data and materials will be made available in compliance with ethical and legal requirements, and any restrictions on access to the data will be disclosed to interested parties. The datasets generated and/oor analysed during the current study are available in the “INFO DENGUE” repository https://info.dengue.mat.br/ and “Agritempo” https://www.agritempo.gov.br/agritempo/jsp/PesquisaClima/index.jsp?siglaUF=BA.
